# Hypnosis for Symptom Management in Adult Cancer Patients: What is the Evidence?

**DOI:** 10.1007/s11864-023-01168-y

**Published:** 2024-01-04

**Authors:** Petra Vayne-Bossert

**Affiliations:** Geneva University Hospitals, Hôpital de Bellerive, 11 Chemin de La Savonnière, 1245 Collonge-Bellerive, Switzerland

**Keywords:** Hypnotherapy, Hypnosis, Cancer, Symptoms, Procedure-related symptoms, Anxiety, Distress

## Abstract

As a palliative care specialist and a hypnotherapist, I use therapeutic communication and conversational hypnosis daily in my patient – doctor relationship. Formal hypnotherapy sessions are integrated in my practice whenever patients are open or wish for such an approach in relation to a specific symptom, for better overall management of their disease burden and/or enhanced well-being. Although hypnosis has been used for centuries in medical practice and for thousands of years in healing practices in ancient cultures all over the world, the evidence remains scarce. Nevertheless, in the last 10 years several randomised controlled trials have been conducted, building up an evidence base. In contrast to most oncological treatments, hypnotherapy is far from being considered evidence-based “standard care”. It is however, if practiced by a trained health care professional, almost free of side effects and therefore potentially has a very favourable benefit-to-harm ratio. The question arises whether hypnotherapy will ever become a standard of care intervention? This seems unlikely since its efficacy may be influenced by the patient’s belief in hypnosis and compliance to therapy. Furthermore, a fundamental necessity is a personalised approach that moves hypnotherapy more into the category of individual-centred care rather than standard care.

## Introduction

Cancer patients suffer from various symptoms, some of which will not respond sufficiently to conventional medicine. There is a growing demand from patients for complementary approaches to these problems. Hypnosis is one of these approaches which has gained interest in the last decade and which has become increasingly subject to research studies and trials.

The beginning of hypnosis in Western Europe is historically associated with Franz Anton Mesmer (1734 – 1815), the founder of “animal magnetism” which he practiced in Paris. He used the hypnotic state to realign an invisible magnetic fluid, (that he believed existed in all living things), to treat diseases. His unorthodox group sessions were quite spectacular, highlighted with an orchestra playing music in the background. A century later, Milton Erickson (1901 – 1980), a US citizen, developed modern medical hypnosis on which today’s therapies are still based. He was a master in using language in a very creative way and the founder of what we now call “permissive hypnosis” in contrast to the previously performed “authoritarian hypnosis” (which is still used in performance hypnosis, for example).

But what exactly is hypnosis? There is no clear or unique definition. It is important to explain, that it is **NOT** a state of sleep and the participant does **NOT** lose control. It is certainly NOT magical nor mystical, but a natural state of mind into which everybody enters several times a day. It may be experienced as daydreaming, when we do something on “auto-pilot”, or the state just before drifting off to sleep. This state corresponds mainly to alpha waves in the electroencephalogram (EEG) and has been proven to be the major brain wave seen during meditation and relaxation [1]. The hypnotic state has also been correlated with brain waves. While being in a hypnotic trance state, the EEG measures either alpha waves (light trance) or theta waves (deep trance) which is different from the awakening state (gamma or beta waves) as well as sleep (delta waves) [2]. Hypnosis is an altered state of consciousness in which the participant is focusing and funnelling all their attention and concentration on an image, activity, sensation, sound or a mixture of these sensory experiences. This state of mind, which is also called a “trance”, favours the dissociation of the conscious (analysing mind) and the subconscious. The latter is, during hypnosis, open to suggestions which are channelled by the hypnotherapist in a positive, comprehensive and tailored way to the participant. Due to this state of enhanced attention, the participant experiences his or her symptoms or difficulties in another reality, inducing a “spark” of change, captured by the subconscious, without the interference of the conscious, analytical mind. The continuity of the change will then take place subconsciously, but can be underpinned by “anchor techniques” and easily made available to the patient by post-hypnotic suggestions.

A typical hypnotherapy session, guided by a trained health care professional, starts with the establishment of a confident relationship, history taking (specific to hypnosis), the induction of the trance and the creation of a “safe place”, followed by a series of positive suggestions introduced by the hypnotherapist in relation to the patients’ objectives for the session. The session ends with anchor techniques and post-hypnotic suggestions before the patient is guided back to their normal (conscious) state.

In summary, hypnosis is not a magical pill one swallows, and all the “pain” disappears immediately. It rather helps (re)activate the patient’s own capacities for getting better in a way that is feasible for them. It is a very powerful tool for patient empowerment and the patient can even take ownership of it by learning self-hypnosis.

In order to understand better in what circumstances and for which symptoms hypnosis may be used in patients suffering from cancer, this paper provides a critical review of the literature. The quality of the studies and systematic reviews was not assessed. Nevertheless, it provides general practitioners with information they can use to help guide any cancer patients who inquire about hypnotherapy.

## Anxiety and distress

Anxiety and distress may be felt at any stage following a cancer diagnosis. This may hinder the pursuit of treatment or even prevent patients from receiving curative cancer therapy. Although anxiolytic drugs may be of some help to some patients, they should be avoided if possible, to enable complementary approaches to be tried. Hypnosis is one of these approaches which has shown to be of use in several studies. Chen et al. found in their comprehensive meta-analysis including RCTs and pre-post-design studies [3], that hypnosis significantly improved the participant’s immediate anxiety compared to standard care, attention care, distraction or cognitive-behaviour-therapy (CBT). Furthermore, the effect was maintained over time (from 1 to 6 months after the intervention) with particular benefit in paediatric patients and patients with haematological malignancies undergoing procedures such as lumbar puncture, bone marrow aspiration or venipuncture. Hypnotherapy guided by a therapist was shown to be more helpful than self-hypnosis.

## Procedure-related symptoms

Any surgical intervention is associated with a certain degree of anxiety and distress, procedure-related pain or prolonged pain post-intervention, as well as nausea and/or vomiting. The impact of hypnosis on any of these symptoms is one of the best studied areas, particularly in patients with breast cancer, where recent trials with adequate sample sizes have been published. A summary of the studies is listed in Table 1.

**Table 1:** Randomised controlled trials on efficacy of hypnosis on procedure-related symptoms in cancer patients

Population	Procedure	Hypnosis intervention	Control intervention	Outcome summarised
Breast cancer patients *N* = 236 [4]	Core needle image-guided biopsy	Standard hypnotic script read to the patient during procedure	• Standard care • Empathic attention	Anxiety: increased in standard care group, stayed the same in empathic attention and was decreased in hypnosis Pain: increased significantly in all groups
Breast cancer patients *N* = 150 [5] *N* = 200 [6] *N* = 90 [7]	Minor breast cancer surgery or excisional biopsy or lumpectomy	15-min hypnosis session or less before general anaesthesia or surgery	• Standard care • Empathic listening	Pain: inconsistent results on pain, low quality Sedatives: Hypnosis may lower the requirements of propofol, lidocaine and opioids Fatigue: significantly lower scores in hypnosis Nausea: significantly lower scores in hypnosis Emotional upset: significantly lower scores in hypnosis Anxiety: significantly lower scores in hypnosis Relaxation: significantly higher levels of relaxation in hypnosis
Various haematological malignancies *N* = 80 [8]	Bone marrow biopsy	Hypnosis during the procedure	Standard of care	Pain: no statistical significant difference although the pain scores were slightly lower in hypnosis group Anxiety: statistically significant greater reduction in hypnosis group
Haematological malignancies *N* = 67 [9]	Bone marrow transplant	Extensive hypnosis training	• Cognitive behavioural coping skills • Usual care • Therapist contact	Pain: statistically significant lower oral pain in hypnosis group, but very few patients in each arm (10–12) Nausea, vomiting or opioid use: no difference
Benign tumours (mostly uterine fibroids) and hepatic malignancies *N* = 201 [10]	Percutaneous tumour embolisation or radiofrequency ablation	Hypnosis group vs standard care vs attention		Less pain and anxiety Less midazolam or fentanyl (33% less compared to standard care and 43% less compared to empathic attention)
Breast cancer patients *N* = 167 [11]	Marker placement under radiographic control prior to breast cancer surgery	Conversational hypnosis	Standard care	Anxiety: the study was prematurely interrupted for futility, absence of statistically significant difference after accrual of 167 patients

In a large observational study involving 300 patients in a Belgium Breast Cancer Clinic, women were asked before breast surgery whether they wanted to participate either in a standard general anaesthesia group or in a hypno-sedation group (without general anaesthesia). One hundred and fifty consecutive participants were included in each group and compared according to several outcomes. The hypno-sedation group had a statistically significant shorter duration of hospitalisation, needed less post-mastectomy lymph drainage and reported less anxiety in the post-operative period. Furthermore, the effect was sustained, and asthenia was also decreased during subsequent adjuvant therapy. Despite the fact that it was not a randomised trial, the results highlight several benefits including avoidance of general anaesthesia [12].

## Radiotherapy and hypnosis

In 2005, Staplers et al. included 69 patients in a trial where patients were randomised either to the hypnosis group (several sessions before and during radiotherapy) or to standard care (radiotherapy alone). The major endpoints were anxiety and quality of life neither of which benefitted from hypnosis. However, mental health and overall well-being were superior in the intervention group [13].

Pain was the main outcome measured in patients with head and neck cancer undergoing radiotherapy. This study showed a statistically significant benefit in the hypnosis group versus usual care [14].

Radiotherapy is often accompanied by intense fatigue. Montgomery et al. randomised 42 women with breast cancer before radiotherapy either to a combined intervention group (hypnosis with CBT) or to standard care. Fatigue measured over time was the main end point. This remained unchanged in the intervention group, whereas the control group experienced increasing fatigue over the treatment period [15].

In a study by Schnur and colleagues, hypnosis combined with CBT was supportive of a positive emotional state in women during radiotherapy treatment [16].

## Cancer pain

Pain is very frequent not only in patients with active cancer, but also in cancer survivors and has a high negative impact on the quality of life. Two thirds of patients with advanced disease, up to 55% receiving cancer treatment and around 40% of cancer survivors have reported pain [17]. There are various mechanisms implicated in the pain perception, such as nociceptive and neurological damage directly caused by the cancer or by cancer treatment or surgery.

Most of the studies that tested hypnosis in cancer patients experiencing pain were carried out in procedural, surgical or radiotherapy-related pain situations as discussed above. For this reason, the recently published Society for Integrative Oncology (ASCO) Guidelines only recommend hypnosis for procedural or surgical pain in adult cancer patients and preferably with hypnosis provided during the whole intervention and not as a single pre-intervention session or self-hypnosis. These guidelines are based on five RCTs, graded as intermediate in quality of evidence and with only a moderate strength of recommendation. For other types of pain, the evidence is either too weak to be recommended or still needs to be demonstrated in controlled clinical trials [18••].

In a systematic review and meta-analysis on mind–body therapies and cancer pain in adults, conducted in 2022 [19•], three studies were included, but only the two older studies [20, 21] included cancer patients with pain that was not procedure related. The first study used very limited hypnosis (only 5–10-min self-hypnosis exercise at the end of a 90-min group therapy) and only in a subset of the intervention group. Although the intervention group with hypnosis had better pain control during a 1-year follow-up, the sample size was very small (*N* = 19) (12). Ebell used a cross-over design which is questionable for hypnosis as a carry-over effect cannot be excluded [21].

## Cancer-related fatigue

Cancer-related fatigue is often a difficult symptom to treat. There are no specific medical treatments that have shown convincing results [22]. Various complementary approaches as well as a healthy lifestyle may be more promising in improving fatigue. Several trials have been undertaken including hypnosis as a part of a multidimensional approach. There are no studies testing hypnosis as a sole intervention.

A randomised controlled study with a small sample size (*N* = 44) involving patients with various cancers demonstrated that hypnosis combined with CBT was significantly more effective in improving fatigue than the control discussion only group [23].

Grégoire et al. have studied cancer-related fatigue and the impact of hypnosis in several clinical trials. In a non-randomised multiple arm trial that included 114 patients with non-metastatic breast cancer, patients were offered to participate in any of the three groups: yoga, self-hypnosis or CBT. A fourth control group was formed with the 24 patients who declined to participate. Outcomes were measured at 9 months after the intervention and showed a sustained decrease in fatigue, anxiety and depression scores in the hypnosis group. Only the yoga group also reported lower anxiety scores. CBT intervention had no impact on any of the symptoms measured [24].

Results of another trial by Grégoire et al. were published in 2021 [25••]. Ninety-five patients with breast cancer were randomly assigned to either an 8-week group intervention combining self-hypnosis training and self-care instructions or to a waiting list group. This study showed a significant immediate decrease in emotional distress and improvement in insomnia. A secondary analysis including data on the same symptoms 1 year later proved that the positive effect of the combined intervention was sustained over this time period [26••].

## Anticipatory or chemotherapy-induced nausea and vomiting

The most recent systematic review was conducted by Richardson et al. in 2007 and included six randomised controlled studies (RCT), but only one study included adult patients with cancer [27]. In this four-arm study (hypnosis versus cognitive coping versus therapist attention versus usual care), there was no statistically significant difference in nausea or vomiting among the 67 patients included. Since then, only one additional prospective controlled study has been published [28] in women diagnosed with ovarian cancer. Patients were offered to participate in a randomised trial of a combined intervention including hypnosis, therapeutic massage and healing touch during chemotherapy versus chemotherapy alone. There was no effect on the amount of anti-emetic medication used [28].

Despite this very low evidence, some international oncological guidelines, such as MASCC/ESMO consensus recommendations, remain in favour of offering hypnosis for anticipatory nausea and vomiting in children and adults receiving chemotherapy [29].

## Hot flushes in breast cancer

Hot flushes are a very common and disturbing symptom in pre- or postmenopausal women, breast cancer survivors or patients receiving anti-hormonal drugs or chemotherapy. Up to 80% of women have reported hot flushes which are particularly evident following cancer treatment and may have a negative impact on quality of life and sleep [30].

One RCT focuses on symptom control with hypnosis in 60 breast cancer survivors. The intervention group was provided with an intense hypnosis training session including self-hypnosis practices and audio-recordings. In comparison with the control group who received standard care, the study demonstrated that the intervention group not only had an improvement in hot flushes, but hypnosis also had a positive impact on quality of life, sleep, anxiety and depression [31].

## Hypnosis in palliative care

Symptom burden is very high in palliative care patients independent of the underlying disease [32]. Management of these symptoms with medication often results in unpleasant and unwanted side effects. It is reasonable to suggest, therefore, that hypnosis, which is almost entirely devoid of harmful side effects, could be a promising complementary approach in palliative care patients. To date, there have been no RCTs of hypnotherapy undertaken in this population, but a 2-year-long-term follow-up study in chronically ill patients has been published. Two groups have been followed and compared: standard pharmacological care and early integration of clinical hypnosis with self-hypnosis in addition to standard pharmacological care. This cohort study included a total of 50 patients, 25 in each group. Only 13 patients were suffering from advanced cancer, the others had either neurological or rheumatic chronic progressive diseases. Both groups experienced a decrease in pain over a 2-year period, but in the hypnosis group, the decrease was statistically more significant. The control group had a four-times greater risk of increasing opioid use than the intervention group. Furthermore, the hypnosis group suffered from less anxiety after 2 years than the control group who reported unchanged levels [33].

## Safety of hypnosis, adverse events or unexpected reactions

Although hypnosis is almost entirely free of side effects, and very few studies have reported such events, some patients might experience the effects described below. These reactions can be reduced to a minimum with a properly trained and experienced hypnotherapist. They have been reported more often in people hypnotised during a stage performance, than during a therapeutic session [34, 35]. It is common for the patient to experience unfamiliar feelings or physical sensations. Often these are therapeutically useful and part of the dissociation process, but they may generate anxiety and the fear of losing control in some patients. An experienced hypnotherapist knows how to handle these situations and to include them in a positive and therapeutic way [36]. More often, such unexpected reactions develop during psychotherapeutic sessions. Psychologists have indeed reported interruption of relaxation therapy in 3.8% of their patients because of adverse effects [37, 38]. This is much less common when physical symptoms are being treated. Bollinger has published a review of adverse events in clinical studies of hypnosis. He found a rate of 0% of serious adverse events and an average of 0.47% for all other side effects, of which most were dizziness, a sensation of floating or heaviness and headaches [39]. Nevertheless, very few trials even report such events, probably also because there are so far, no standardised methods to assess and report adverse events and side effects of hypnosis.

The most common problem a hypnotherapist may encounter is the difficulty of re-alerting or dehypnotising the patients. Again, a well-trained therapist has learned various techniques to provide a proper and safe response to this situation [40].

## Discussion

This summary of the evidence-base for hypnosis in cancer patients shows that randomised controlled trials have been emerging in recent years, but have not reached the level, (with the exception of procedural and surgical pain), to be considered standard complementary care. Most, especially the older studies, had very small sample sizes, potentially not adequate to provide sufficient power. Research with hypnotherapy remains a challenge in several aspects. Firstly, patients will not be able to be blinded, particularly as the effect of hypnosis is even more profound if the patient experiences clear hypnotic phenomena (e.g. levitation or catalepsy). Secondly, medical hypnotherapy is patient-centred care, and every session is individually tailored to the patient’s perception of the world and their specific needs). Standardised hypnotherapy sessions (e.g. script or audio-tapes) will never have the same impact as a personalised intervention, and efficacy may, therefore, be difficult to prove. Thirdly, as with other psychological interventions, not every patient is enthusiastic to receive such an approach, and with limited compliance, hypnotherapy will be less efficient or not work at all.

Last, but not least, hypnotherapy, if practiced by a trained health care professional, has a very favourable benefit-to-harm ratio which is not the case with most of the standard medical treatments used for symptom control. Even in the absence of clear evidence of benefit, it seems possible that hypnotherapy may help cancer patients with symptom management and quality of life, but more rigorous evaluation is required.
